# Generative dynamical models for classification of rsfMRI data

**DOI:** 10.1162/netn_a_00412

**Published:** 2024-12-10

**Authors:** Grace Huckins, Russell A. Poldrack

**Affiliations:** Neurosciences Interdepartmental Program, Stanford University, Stanford, CA, USA; Department of Psychology, Stanford University, Stanford, CA, USA

**Keywords:** Resting-state fMRI, Hidden Markov models, Classification, Generative models, Network dynamics

## Abstract

The growing availability of large-scale neuroimaging datasets and user-friendly machine learning tools has led to a recent surge in studies that use fMRI data to predict psychological or behavioral variables. Many such studies classify fMRI data on the basis of static features, but fewer try to leverage brain dynamics for classification. Here, we pilot a generative, dynamical approach for classifying resting-state fMRI (rsfMRI) data. By fitting separate hidden Markov models to the classes in our training data and assigning class labels to test data based on their likelihood under those models, we are able to take advantage of dynamical patterns in the data without confronting the statistical limitations of some other dynamical approaches. Moreover, we demonstrate that hidden Markov models are able to successfully perform within-subject classification on the MyConnectome dataset solely on the basis of transition probabilities among their hidden states. On the other hand, individual Human Connectome Project subjects cannot be identified on the basis of hidden state transition probabilities alone—although a vector autoregressive model does achieve high performance. These results demonstrate a dynamical classification approach for rsfMRI data that shows promising performance, particularly for within-subject classification, and has the potential to afford greater interpretability than other approaches.

## Author Summary

Neuroimaging researchers have made substantial progress in using brain data to predict psychological and behavioral variables, like personality, cognitive abilities, and neurological and psychiatric diagnoses. In general, however, these prediction approaches do not take account of how brain activity changes over time. In this study, we use hidden Markov models, a simple and generic model for dynamic processes, to perform brain-based prediction. We show that hidden Markov models can successfully distinguish whether a single individual had eaten and consumed caffeine before his brain scan. These models also show some promise for “fingerprinting,” or identifying individuals solely on the basis of their brain scans. This study demonstrates that hidden Markov models are a promising tool for neuroimaging-based prediction.

## INTRODUCTION

Historically, human neuroimaging research focused on uncovering associations between brain measures and psychological variables. In recent years, however, parts of the field have pivoted away from “mere association” and toward predicting psychological and behavioral measures using brain data. Two concurrent developments have enabled this shift: the availability of software that allows researchers to straightforwardly deploy machine learning methods and the release of numerous large-scale fMRI datasets, which allow for those methods to be effectively deployed on high-dimensional neuroimaging data.

Because resting-state fMRI (rsfMRI) data ostensibly represent an individual’s baseline brain activity in the absence of a contrived task, they have frequently been used to predict relatively stable attributes, like personality traits (Feng et al., 2018; Liu, Kohn, & Fernández, 2019), cognitive ability (Dubois, Galdi, Paul, & Adolphs, 2018; Wen, Liu, & Hsieh, 2018), neurological disease (Ju, Hu, Zhou, & Li, 2019; Saccá et al., 2019), and psychiatric diagnosis (Plitt, Barnes, & Martin, 2015; Wang, Zhou, Gui, Liu, & Lu, 2023; Yoshida et al., 2017). rsfMRI-based prediction is especially well-represented in the biological psychiatry literature, where it is deployed both to predict treatment outcomes and to classify scans according to diagnostic labels (Bondi, Maggioni, Brambilla, & Delvecchio, 2023; Di Martino et al., 2014; HD-200 Consortium, 2012; Santana et al., 2022; Taspinar & Ozkurt, 2024).

In general, rsfMRI-based prediction studies deploy the “discriminative” approach to classification. Under the discriminative approach, models are trained to predict the odds than an observation that belongs to a particular class given the observed data, *p*(*C*_*i*_∣*x*). However, there is also an alterantive approach, called generative classification: Rather than predicting the odds of class membership given data and then assigning a class label on that basis, generative models predict what data should look like given a class label—*p*(*x*∣*C*_*i*_). For example, instead of being trained to determine whether an rsfMRI recording comes from someone with ADHD (attention-deficit/hyperactivity disorder) or a healty control, a generative model is trained to simulate an rsfMRI recording from an individual with ADHD (or a recording from a healthy control). By training separate generative models on different classes of training data and then asking which model was more likely to have generated a particular test datum, generative models can be straightforwardly deployed for classification.

The generative approach comes with some important advantages. Because generative models directly simulate fMRI data, they can make use of the dynamic structure of the data while avoiding the statistical limitations of some other approaches. Some researchers have previously used dynamical rather than static features for classification of fMRI (e.g., Ikeda, Kawano, Watanabe, Yamashita, & Kawahara, 2022; Jun, Kang, Choi, & Suk, 2019; Thome, Steinbach, Grosskreutz, Durstewitz, & Koppe, 2022), and one study that directly compared static and dynamic features found that the latter were far more effective for distinguishing individuals with schizophrenia or bipolar disorder from healthy controls (Rashid et al., 2016). In general, however, such studies remain in the minority. Furthermore, studies that do use dynamic measures as a substrate for classification often deploy the sliding window approach, and the results obtained from using this technique are sensitive to the size of the window (Shakil, Lee, & Keilholz, 2016). Because of the statistical limitations of the sliding window approach, there has been interest in developing alternative techniques for capturing fMRI dynamics that do not require the use of sliding windows, such as phase-based approaches (Deco & Kringelbach, 2016; Glerean, Salmi, Lahnakoski, Jääskeläinen, & Sams, 2012). The generative model approach similarly avoids this limitation.

Moreover, the generative approach has the potential to afford greater interpretability than other dynamical approaches, taking into account that dynamics can increase the number of features available for discriminative classification by orders of magnitude. There are methods for identifying the most important features, but there is no guarantee that this set will be small enough to allow for interpretability. The generative approach does not guarantee interpretability either, but if generative models with small numbers of parameters are able to successfully classify rsfMRI data, those models may have a distinct interpretability advantage over some discriminative approaches.

In this study, we focus on a simple, flexible class of generative model: the hidden Markov model (HMM). In human neuroscience, HMMs have been used to model both task fMRI and rsfMRI data. Using task data, Baldassano et al. (2017) applied HMMs to the detection of narrative event boundaries, and Taghia et al. (2018) used an extension of the HMM known as a switching linear dynamical system to identify latent brain states in the *n*-back task. Using resting-state data, Vidaurre, Smith, and Woolrich (2017) deployed an HMM to identify two brain activity “metastates” between which subjects transitioned over the course of a scan, and Scofield, Johnson, Wood, and Geary (2019) used HMMs to uncover latent states in resting-state data and found that state occupancies and transitions were associated with both sex and ADHD diagnosis.

Fewer studies have directly used HMMs to classify fMRI data, although some progress has been made in this direction. HMMs have been successfully deployed to classify scans as pre- or post-transcranial magnetic stimulation (Bustamante, Castrillón, & Arias-Londoño, 2023) and to distinguish subjects with mild cognitive impairment (Suk, Wee, Lee, & Shen, 2016), post-traumatic stress disorder (Ou et al., 2015), and ADHD (Dang, Chaudhury, Lall, & Roy, 2017) from healthy controls. One particularly promising study from Nielsen et al. (2018) demonstrates that HMMs with simple emission distributions and small numbers of hidden states can distinguish individuals with schizophrenia and health controls with between 70% and 75% accuracy.

However, some other studies use a high number of hidden states (>20), a multi-HMM voting procedure, complex emission models, or nontransparent dimensionality reduction procedures (e.g., autoencoders), all of which limit interpretability. Moreover, to our knowledge, no previous studies have attempted to use HMMs for generative classification of fMRI data solely on the basis of hidden-state transition probabilities. It is therefore necessary to more thoroughly and robustly evaluate the utility of HMMs, especially HMMs with a small number of hidden states, for rsfMRI-based classification.

Here, we pilot a generative, interpretable approach to rsfMRI classification. We have chosen two very different classification tasks on which to test our approach: a within-subject binary classification task and a between-subject fingerprinting task. For the first task, we attempt to distinguish days on which a single subject was fed/caffeinated versus fasted/uncaffeinated; these caffeinated and uncaffeinated recordings have been previously shown to exhibit different whole-brain connectivity patterns (Laumann et al., 2015). For the second, we attempt to identify the specific individual from whom a resting-state scan originated, out of a group of 100 potential subjects, a task known as “fingerprinting” that has previously been solved with near-perfect accuracy using functional connectivity matrices (Finn et al., 2015).

## RESULTS

### Within-Subject Binary Classification Performance

We first evaluated the performance of the HMM approach by training it to classify fed/caffeinated versus unfed/uncaffeinated runs in the MyConnectome dataset (henceforward referred as “caffeinated” and “uncaffeinated” for simplicity). Multiple variations of HMM-based classification were tested on this classification task: The models used either diagonal Gaussian or autoregressive emission distributions, and models were either fully trained or trained using a transition matrix-only (“trans-only”) regime, in which all other model parameters were held constant. The observations used to train the models were network activation data, determined using either the 7- or 17-network Yeo et al. parcellation (Yeo et al., 2011). A support vector machine (SVM) trained on the network-level functional connectivity matrices for each run was used as a baseline model. Model performance was first evaluated using cross-validation; performance was then assessed using a held-out subset of the data. We used held-out datasets throughout this paper in an attempt to ensure the replicability of our results, and we evaluated model performance on those held-out data only after the approach and implementation had been finalized.

**Figure 1.**
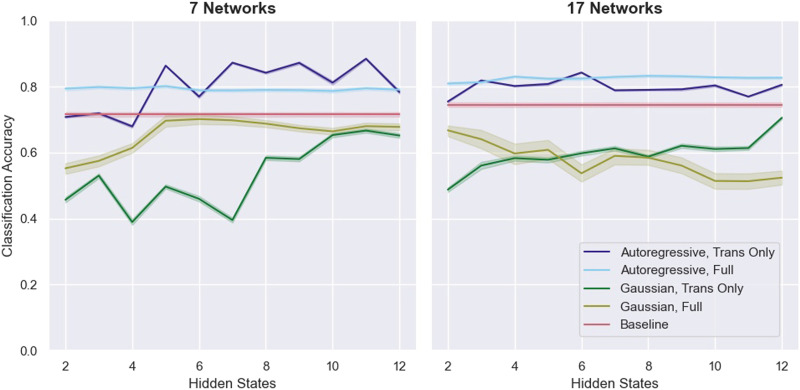
The classification performance of various HMM-based approaches on the MyConnectome dataset. Results using the Yeo et al. (2011) 7-network parcellation are shown on the left; results using the 17-network parcellation are on the right. Error bars are 95% confidence intervals over 100 runs of cross-validation. Chance performance is 50%.

In cross-validation, HMM classification using a diagonal Gaussian emission model consistently underperformed the baseline SVM classifier (Figure 1). The fully trained Gaussian HMM did exceed chance performance on the 7-network data, but it approached chance performance on the 17-network data for large numbers of hidden states. In contrast, for small numbers of hidden states, the transition matrix-only approach failed to exceed chance performance, particularly on the 7-network data, but as the number of hidden states increased up to 12, it did approach baseline performance.

On the other hand, HMM-based classification with an autoregressive emission model consistently outperformed the baseline approach. The fully trained autoregressive HMM (ARHMM) approach achieved around 80% accuracy regardless of the number of hidden states. The transition matrix-only approach achieved similar levels of performance, as long as the number of hidden states exceeded some minimum threshold (five states for the 7-network data and three states for the 17-network data). In fact, for the 7-network data, the transition matrix-only ARHMM approach generally exceeded the performance of the fully trained ARHMM approach.

The similar performance of the transition matrix-only and fully trained ARHMM approaches suggests that the success of the ARHMM classification approach was driven by dynamical features of the data, features that are lost when static measures like functional connectivity are used. Dynamical information alone—in this case, the probability distribution of transitions between hidden states—is adequate to distinguish uncaffeinated from caffeinated resting-state recordings in the MyConnectome dataset.

In a regression analysis, with classification accuracy as the outcome variable, model type and number of hidden states were highly significant regressors (*p* < 0.001), and the number of networks was marginally significant (*p* = 0.07) (Supporting Information Table S1). It is worth nothing that since these *p* values were calculated over many simulation repetitions, they would be expected to be quite high, even for small effect sizes, so they should not be overinterpreted.

**Figure 2.**
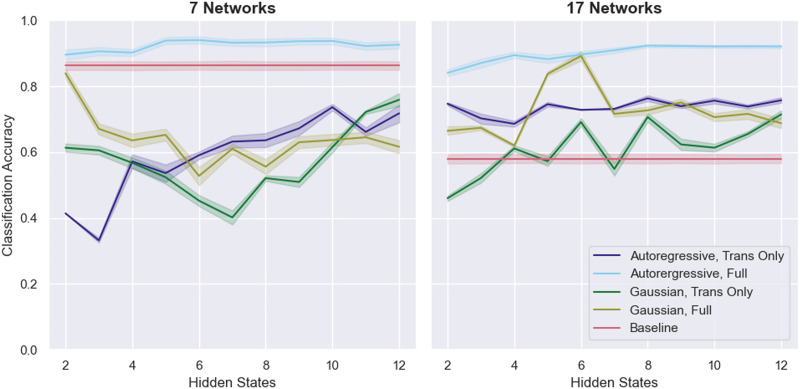
The classification performance of various HMM-based approaches on the 12 held-out runs from the MyConnectome dataset. Error bars are 95% confidence intervals over 100 runs of testing.

Surprisingly, the external validation results on the 12 runs of held-out data show a different pattern from the cross-validation results, although this difference may reflect the small size of the held-out dataset (Figure 2). While the fully trained ARHMM performs even better than it did in cross-validation, reaching a maximum accuracy of 94.0% with seven networks and six hidden states, the transition matrix-only ARHMM does notably worse, reaching a peak performance of 76.4% with 17 networks and eight hidden states. All models except for the fully trained ARHMM exhibit variable performance. Unlike in the cross-validation results, the fully trained Gaussain HMM sometimes rivals the performance of the ARHMM and attains a maximum accuracy of 89.2% with 17 networks and six hidden states.

Notably, the baseline SVM model also exhibits results that diverge from its cross-validation performance. Whereas it achieved 71.5% and 74.5% cross-validated accuracy on the 7-network and 17-network data, respectively, it does substantially better on the held-out 7-network data (86.3%) and substantially worse on the held-out 17-network data (58.0%). The high variability in these results is likely a product of the small size of the validation set.

### Model Features Driving Binary Classification Performance

**Figure 3.**
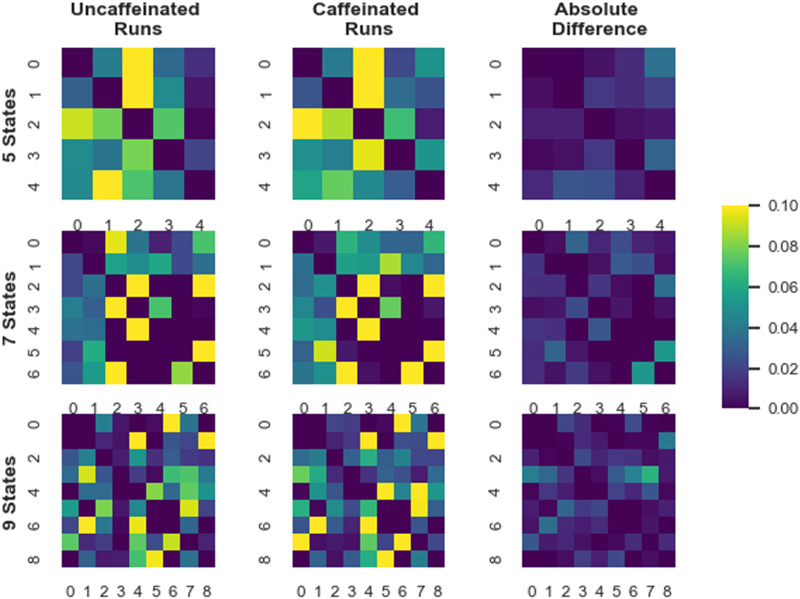
Transition matrices from ARHMMs fit to both uncaffeinated and caffeinated datasets, as well as the absolute value of their difference. Results are shown for 5-, 7-, and 9-state ARHMMs. Rows correspond to the origin states (i.e., the state from which the HMM is transitioning), and columns correspond to the destination states (i.e., the state to which the HMM is transitioning). States are zero-indexed, for example, for the 5-state model, states have been assigned numbers 0–4. Elements along the diagonal—that is, the probability of remaining in a given hidden state—have been set to 0 for visualization purposes.

Given the success of transition matrix-only ARHMM classification on the MyConnectome dataset, we next sought to identify the features of the underlying HMMs that might underpin the performance of that approach. Because only the transition matrices differ between the trans-only ARHMMs fine-tuned on the uncaffeinated and caffeinated subsets of the data, we started by looking directly at those matrices. As the 5-, 7-, and 9-state ARHMM trans-only models performed particularly well on the 7-network data, we decided to specifically interrogate these three models in more detail (Figure 1). We directly compared transition matrices for models fit to all of the uncaffeinated data with transition matrices for models fit to all of the caffeinated data. Figure 3 shows those transition matrices, as well as the absolute differences between them. Some patterns are apparent: In the 5-hidden-state models, the clearest differences are the probabilities of transitioning to or from hidden State 4, and the clearest differences in the 9-hidden-state model are the probabilities of transitioning from hidden State 3. For the 7-hidden-state model, the most apparent differences are the probabilities of transition between States 5 and 6. (It is important to note that the numbering of the hidden states is arbitrary, and hidden states with the same number in models of different dimensionalities do not have any particular relationship to each other.)

**Figure 4.**
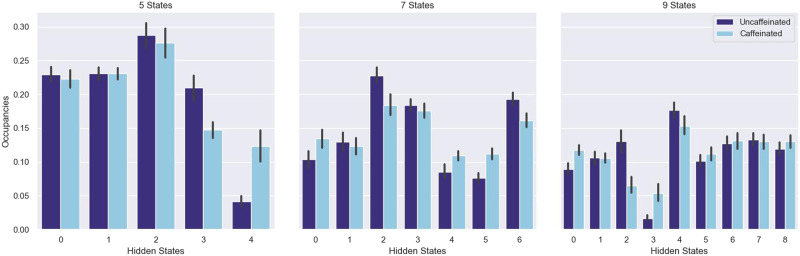
The fraction of time spent in each hidden state (“state occupancy”) for the 5-, 7-, and 9-state ARHMMs. Error bars are 95% confidence intervals computed across the state occupancy times for the 33 uncaffeinated and 26 caffeinated runs.

We also investigated the state occupancy times for the uncaffeinated and caffeinated runs (Figure 4). We determined the state occupancy by calculating the maximum likelihood sequence of hidden states for each run of observations under the fit ARHMMs. In general, the occupancy time data align with the transition matrix data: State 4 again shows large differences in the 5-hidden-state model, and State 3 shows differences in the 9-hidden-state model. However, the occupancy time distributions also point to some features that are not evident in the transition matrices: We see large differences in State 3 in the 5-state model, and in numerous hidden states in the 7- and 9-state models.

**Figure 5.**
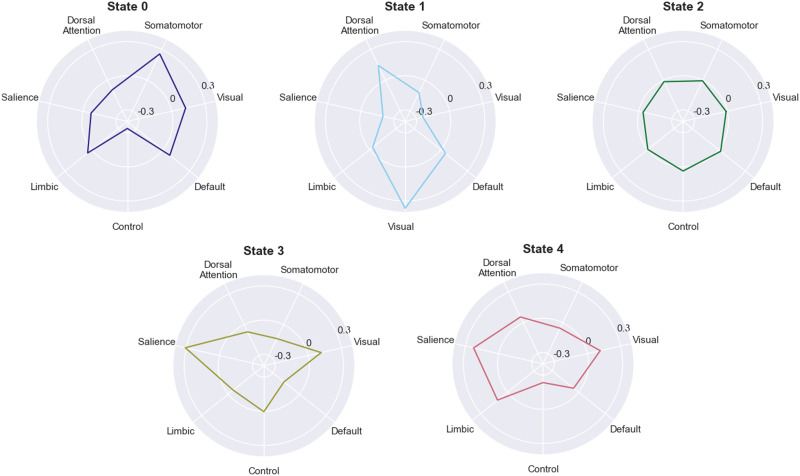
The average network activity for each hidden state in the 5-state ARHMM. The radius of each plot represents the average *z*-scored activity of the network in each state across all caffeinated and uncaffeinated runs.

Finally, we investigated the activity patterns associated with each hidden state in the five-state, 7-network model. We decided to focus on this model in particular because it balances high performance with a low number of hidden states, which enhances interpretability. There are some evident patterns: State 2, for example, can be cautiously interpreted as a “neutral” state in which all networks have near-average activity levels (Figure 5). Additionally, it is suggestive that the whole-brain network activation patterns whose occupancies differ for the “caffeinated” and “uncaffeinated” runs—State 3 and State 4—both involve prominent activation of the salience network. These differences could potentially correspond to the effect that caffeine had on the subject’s engagement with the (limited) external stimuli present during the resting-state scans, although that idea is, at this point, highly speculative.

### Head Motion Does Not Drive Binary Classification Performance

In the data used to obtain the previously reported classification results, volumes with high head motion had been scrubbed and interpolated over. It is therefore possible that differences in the head motion between caffeinated and uncaffeinated recordings may have been driving the results, as opposed to differences in functional brain data. To account for this possibility, we directly compared the classification performance on a dataset that included the interpolated volumes and a dataset from which high-motion volumes had been censored, without interpolation. In both cases, the models were fit to temporally contiguous sequences of volumes: In the censored dataset, each stretch of low-motion data was treated like a separate run, so as not to violate the temporal continuity assumed by the HMM.

**Figure 6.**
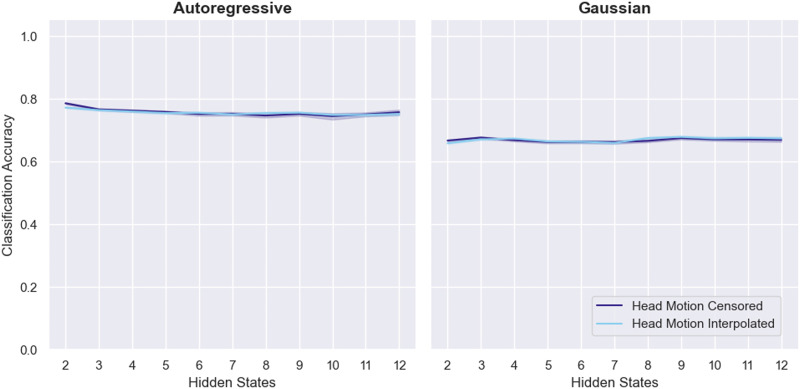
Classification performance on the MyConnectome dataset with high-motion volumes simply censored (“Head Motion Censored”) or with high-motion volumes censored and then interpolated over (“Head Motion Interpolated”). The left panel shows results from the ARHMM-based approach and the right from the Gaussian HMM. All models were fully trained. Chance performance is 50%.

The results of this analysis are shown in Figure 6. Interpolation appears to have no effect on performance, which provides some evidence that head motion differences do not drive the success of this classification approach. Additionally, a linear SVM trained to classify runs as caffeinated versus uncaffeinated based on the number of high-motion volumes in each run achieved an averaged cross-validated balanced accuracy of 48.5%, compared with a chance level of 50%.

### Between-Subject Fingerprinting Performance

**Figure 7.**
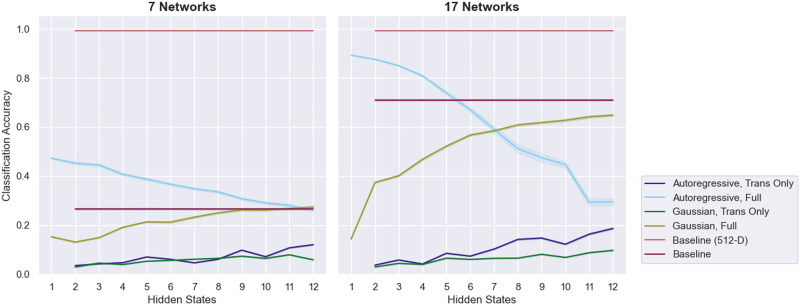
The fingerprinting performance of various HMM-based approaches on the HCP dataset. Results using the Yeo et al. (2011) 7-network parcellation are shown on the left; results using the 17-network parcellation are on the right. Baseline fingerprinting results evaluated on the 512 ROI data (“Baseline (512-D)”) are shown in both panels as a performance ceiling, whereas the individual “Baseline” results in each panel were computed using 7- and 17-network data, respectively. To evaluate performance, models were trained using a “leave-one-run-out” regime—models were trained on three runs from an individual and tested on the fourth. Error bars are 95% confidence intervals over 100 repetitions of training and testing on randomly selected subsets of 100 subjects from the core dataset of 149 subjects. Chance performance is 1%.

Next, we tested the HMM-based classification pipeline on the more challenging task of fingerprinting individuals from the Human Connectome Project (HCP) dataset using their resting-state data. The baseline “correlation of correlations” approach (Finn et al., 2015) showed near-perfect performance when 512 region of interest (ROI) data were used, but the performance of that approach was substantially lower on the 7- and 17-network data (especially the former) (Figure 7).

The fully trained Gaussian HMM mostly fell short of baseline performance, although it did approach baseline performance as the number of hidden states increased toward 12 and the model gained additional degrees of freedom. On the other hand, the ARHMM approach performed better than the 7- and 17-network baseline approaches for low numbers of hidden states, and its performance fell as the number of hidden states increased, potentially due to overfitting. In fact, the best-performing ARHMM approach was a degenerate ARHMM with only a single hidden state, equivalent to a vector autoregressive model. On the 17-network data, this vector autoregressive approach achieved approximately 90% accuracy, compared with a chance level of 1%.

Compared with the fully trained Gaussian and ARHMM approaches, the transition matrix-only approaches performed markedly worse. Although performance did increase with the number of hidden states for the ARHMM transition matrix-only approach, it peaked around 10% accuracy on the 7-network data and 20% accuracy on the 17-network data, far below baseline performance. However, the approaches did consistently exceed chance performance.

In a regression analysis, with classification accuracy as the outcome variable, model type and number of networks were highly significant regressors (*p* < 0.001), and the number of hidden states was marginally significant (*p* = 0.08) (Supporting Information Table S2). This last result seems to be a product of the fact that the ARHMM and Gaussian HMM showed opposite performance patterns across different numbers of hidden states.

**Figure 8.**
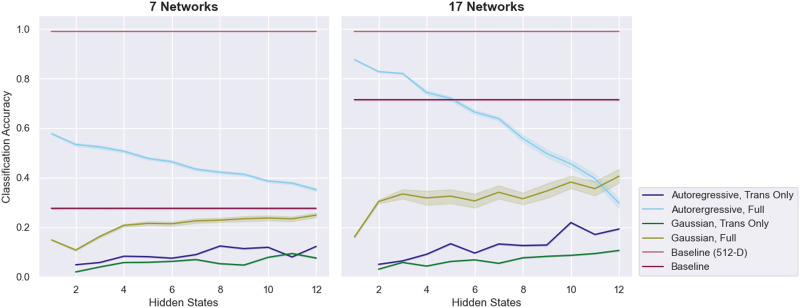
The fingerprinting performance of various HMM-based approaches on the 47 individuals held out from the HCP dataset. Error bars are 95% confidence intervals over 100 runs of testing.

Fingerprinting performance on held-out data largely confirms the patterns observed in cross-validation testing (Figure 8). The only obvious exception is the performance of the fully trained Gaussian HMM on the 17-network data, which is noticeably worse, and more variable, than the performance of that same model in cross-validation testing.

**Figure 9.**
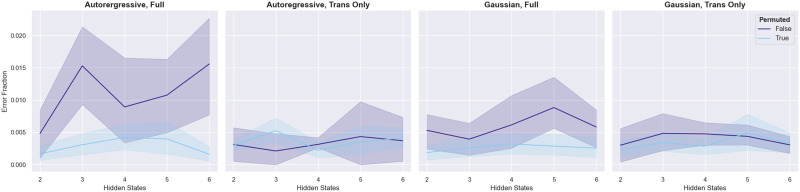
The fraction of misidentification errors that occurred within families, for the four model types, with two to six hidden states. Models were trained with the 7-network data. The blue series indicate the results of the same analysis performed after family membership labels were permuted, in order to create an empirical null distribution. Error bars are 95% confidence intervals over repetitions of model training/testing and, where relevant, family label permutation (5 repetitions for the unpermuted data and 20 for the permuted data).

Finally, because the HCP subjects we selected for this study were not filtered based on family structure, we examined whether our fingerprinting approach was more likely to confuse family members than unrelated individuals. To evaluate this, we calculated the fraction of misidentifications that were made within families, out of the total number of misidentifications. To generate a null distribution under the assumption that family relatedness has no effect on the likelihood of misidentification, we calculated this fraction again after permuting family membership labels. As a whole, family misidentifications were rare; they never constituted above 2% of total misidentifications, regardless of model and number of hidden states (Figure 9). However, for both the fully trained autoregressive and fully trained Gaussian models, family misidentifications were more common than would be expected by chance. This suggests that these two, better-performing models were sensitive to familial similarities in network dynamics. Importantly, neither of the poorly performing, transition matrix-only models seemed affected by family structure. The poor performance of these models therefore cannot be attributed to the challenges introduced by the familial structure of the data.

## DISCUSSION

Overall, these results suggest that the approach presented here—fitting HMMs to training data and classifying test data based on their log likelihood under those models—is a viable method for both within-subject and between-subject classification of rsfMRI data. For both the within-subject MyConnectome classification task and the between-subject HCP fingerprinting task, HMM-based classification compared favorably with baseline, functional connectivity-based techniques. In particular, for both 7- and 17-dimensional network-level data, ARHMM-based fingerprinting outperformed functional connectivity-based fingerprinting, although functional connectivity-based fingerprinting still achieved superior performance when applied to 512-dimensional, ROI-level data. This observation suggests that HMM-based classification may be particularly useful when spatial resolution is limited.

Furthermore, the MyConnectome classification results suggest that dynamical information alone may be adequate for some rsfMRI classification tasks. As long as the number of hidden states was sufficient (at least five for the 7-network data and at least three for the 17-network data), the fully trained ARHMM approach and the transition matrix-only ARHMM approach performed similarly on the within-subject classification task. In the latter case, classification performance is fully attributable to the models’ transition matrices, as those are the only parameters that differ between the “uncaffeinated” and “caffeinated” models. Importantly, these transition matrices, which comprise the probabilities of switching between each pair of hidden states, are abstracted from specific features of the rsfMRI data like network activation and functional connectivity and instead encode high-level information about the dynamics of the data, information that is completely lost when only static metrics are used. Given that the transition matrix-only ARHMM approach, whose performance is completely dependent on dynamic features of the data, does just as well as the fully trained ARHMM approach, whose performance is dependent on both static and dynamic features, it is reasonable to infer that static features play a relatively limited role in the success of the ARHMM-based classification approach in this case.

Moreover, the transition matrix-only approach may afford exciting opportunities for building interpretable dynamical classifiers for neuroimaging data. When classifiers involve many different parameters and depend on a large number of features to achieve high accuracy, it can be challenging to articulate which attributes of the data are driving model performance in a compact, communicable way that can provide insight into neural processes and drive further hypotheses. However, the transition matrix-only approach involves a limited number of parameters by design. Although we did not extensively investigate model parameters in this study, there are already suggestions that the performance of these models may be attributable to a very small number of parameters: The 5-hidden-state, transition matrix-only, ARHMM approach trained on the 7-network MyConnectome data, for example, achieved around 85% accuracy (versus the 50% chance level), but no occupancy differences were observed in Hidden States 0, 1, and 2 in the caffeinated and uncaffeinated recordings (Figure 4). Together with the trained transition matrices (Figure 3), this result suggests that classification performance may be largely attributable to differences in the data in Hidden State 3 and, especially, Hidden State 4. Furthermore, we were able to put forward some tentative hypotheses about the brain basis of these hidden states by examining the average network activation in those states across all runs of data (Figure 5). Future work should strive to make hidden states more meaningful by further investigating their associations with neural variables or even associating them with behavioral variables (although the latter may be difficult if particular hidden states are found to be specific to resting-state data).

Despite its advantages, the transition matrix-only approach may not be a general approach for rsfMRI-based classification tasks. When HMMs were used to label runs of resting-state data from the HCP according to subject (i.e., fingerprinting), transition matrix-only approaches achieved between 5% and 20% accuracy—certainly better than the chance level of 1%, but far worse than baseline approaches. Dynamical information may still be useful for fingerprinting: When basic vector autoregressive models were used to fingerprint the 17-network HCP data, they outperformed the baseline correlation-of-correlations fingerprinting approach. However, these fully trained dynamical models lack the interpretability advantages of the transition matrix-only approach, as even a vector autoregressive model has many more parameters than a transition matrix with a modest number of hidden states. With all of that said, the differences in performance on the MyConnectome and HCP tasks should not be overinterpreted. The datasets were preprocessed according to different pipelines, so the performance differences could, in principle, be attributable to discrepancies in preprocessing rather than the distinct classification tasks and datasets.

It is important to address the results of the validation tests performed on held-out data. For the MyConnectome data, the results on held-out data did partially replicate the results from cross-validation. In both cases, the fully trained ARHMM approach outperformed the baseline SVM approach and showed consistent performance over varying numbers of hidden states. Other results, however, were not replicated. In particular, the transition matrix-only approach performed much worse than the fully trained approach on the held-out data. One explanation for this discrepancy is pure chance. Because the overall dataset was small, the held-out dataset contained only seven uncaffeinated runs and five caffeinated runs. However, it is also possible that the particular methodology used here was effectively “overfit” to the particular sample used over the course of implementation and testing. Although there are no additional data from the MyConnectome project specifically on which the approaches piloted here can be tested, future application of this technique to similar classification tasks should help to distinguish between these two possibilities.

One limitation of this study is the uncertain validity of the model hidden states that appear to underpin performance on the MyConnectome classification task. While these hidden states arguably have potential when it comes to interpretability, especially if models with a small number of hidden states can facilitate successful classification, much more work is necessary to fully realize this potential. In particular, it will be essential in future work to reliably associate the hidden states identified by this approach with meaningful brain and behavioral variables. Ideally, these models will help us uncover global, discrete brain states that are present during rest, but this is not yet assured. One obstacle is the lack of an apparent optimal number *n* of hidden states for the rsfMRI data. In this study, we did not identify an *n* for which our approach consistently exhibited the best performance, and we did not see a clear peak when plotting the cross-validated log likelihood of models with different numbers of hidden states (Supporting Information Figure S1). Together, these results suggest that there is not a specific optimal number of hidden states that applies consistently to all individuals at rest—human brains do not intrinsically move among, say, five discrete hidden states when not engaged in some specific behavior. That said, it may still be possible to link model states with meaningful variables, even if the number of hidden states itself is selected somewhat arbitrarily or varies from individual to individual or from context to context.

However, without any external validation of these hidden states, it is difficult to ensure that certain confounders did not contribute to classification performance. While we did account for the effects of head motion in the MyConnectome classification task, other potential effects of caffeine were impossible to control for. For example, it is possible that the successful classification of uncaffeinated versus caffeinated runs is ultimately a product not of differences in brain activity dynamics but rather of the vascular effects of caffeine, which might conceivably affect the dynamics of blood flow in the brain and, thus, the dynamics of the BOLD signal.

Another limitation of this study, and of the overall approach, is the volume of data needed to successfully train the various HMMs used. Although we had access to high-dimensional, ROI-level data for both the MyConnectome and HCP datasets, training our models on those data was infeasible, because the number of parameters required for the emission distributions would have, in some cases, exceeded the number of fMRI volumes available for training. It was therefore necessary to use network-level summary data instead. In the process of averaging signals over the entire brain networks, a large volume of information was lost—information that may have proven useful for classification. Simultaneously, however, whole-brain dynamical information is lost when static metrics like functional connectivity, which underpin many other classification approaches, are calculated. Choosing a classification approach, and processing data in accordance with that approach, therefore involves intrinsic trade-offs that have to be weighed for every specific problem. It is also worth noting that using network-level data has advantages as well as disadvantages: Low spatial-resolution fMRI data can be obtained more cheaply, and averaging across many ROIs attenuates the impact of region-specific noise.

Ultimately, this study underlines the importance of brain dynamics for within- and between-subject rsfMRI-based classification, especially when spatial resolution is low. For both the caffeinated versus uncaffeinated task and the fingerprinting task, the best dynamical approaches were able to outperform static baselines when applied to 7- and 17-network data. Future research should apply the approach piloted here to different rsfMRI classification tasks—especially group-level classification, which was not attempted here. Additionally, there are numerous extensions of the models used here that might be able to achieve even better classification performance. Switching linear dynamical systems, for example, maintain the same basic Markov backbone, but add to this discrete hidden state a continuous hidden state whose dynamics are dictated by a linear dynamical system. Previous work has used switching linear dynamical systems to predict diagnostic labels based on task fMRI data (e.g., Cai et al., 2021), but, to our knowledge, such models have not previously been applied to rsfMRI data.

By and large, previous approaches to rsfMRI classification have hinged on static features like functional connectivity. Here, we show that dynamical approaches can achieve superior performance on both within-subject and between-subject classification tasks and that, in some cases, purely dynamical features can fully drive successful classification. This study provides support not only for further exploring dynamical classification approaches for rsfMRI data but also for studying dynamics in rsfMRI more broadly, especially when it comes to understanding changes in resting-state brain activity within an individual.

## METHODS

### Data

Two datasets were used for this study. The first, the MyConnectome dataset, comprises 84 rsfMRI scans collected from a single individual (author R.A.P., male, healthy, aged 45 years at the beginning of data collection), all on different days. Scans were performed using a Siemens Skyra 3-T MRI scanner using a multiband EPI sequence with a repetition time of 1.16 s. Each scan lasted 10 min, resulting in 518 volumes per scan. Seventy-one of these scans were performed at the same time in the morning—40 on Tuesday, when the subject had not yet consumed caffeine or food that day, and 31 on Thursday, when he had. Because network-level differences have previously been documented between the Tuesday and Thursday scans (Laumann et al., 2015), these 71 scans were selected for classification analysis. Seven Tuesday (uncaffeinated) and five Thursday (caffeinated) scans were held out for later valdiation analysis, and the remaining 33 uncaffeinated and 26 caffeinated scans were used for initial implementation and testing of the below methods. More details about the participant and the scanning procedure can be found in Laumann et al. (2015).

**Figure 10.**
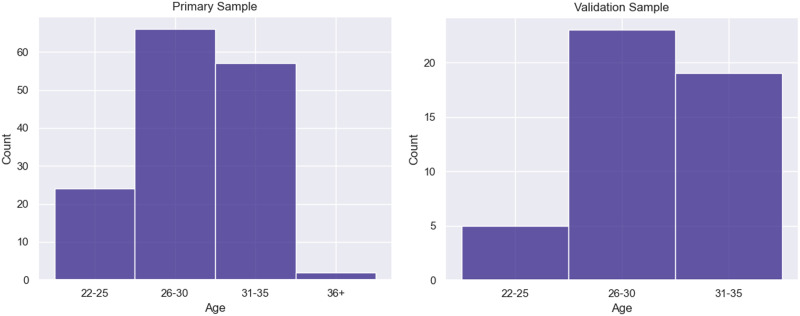
Age distributions for both the primary and validation HCP samples.

The second dataset consists of rsfMRI scans from 196 HCP participants. All HCP data were recorded on a customized Siemens Skyra 3-T MRI scanner at Washington University in St. Louis, using a gradient-echo EPI with a repetition time of 720 ms. Each resting-state scan lasted 14 min and 33 s, yielding a total of 1,200 volumes. The 196 participants used in this study are approximately the first 196 HCP subjects, listed by subject identifier, for whom all task data were present; subjects were disqualified if they did not have four resting-state scans or if any of their resting-state scans did not contain exactly 1,200 volumes. Of the four resting-state scans that each of these subjects had available, two scans were recorded on each of the two different days. Data from a randomly selected subset of 47 subjects (31 female, 16 male) were held out for later validation analysis, and data from the remaining 149 subjects (85 female, 64 male) were used for initial implementation and testing. Age distributions for both samples are shown in Figure 10. Full details of the HCP dataset can be found in Van Essen et al. (2013).

### Preprocessing

#### MyConnectome.

Data were preprocessed as in Laumann et al. (2015). Briefly, data from each session were registered to a single session from the participant, which was registered to an atlas. Nuisance regressors were removed, high-motion volumes were censored and interpolated over, and the data were band-pass filtered (0.009 < *f* < 0.08 Hz). An anatomical surface for the subject was generated using FreeSurfer, and BOLD volumes were sampled to that surface. Based on the full set of recordings, an individualized parcellation was obtained, resulting in 630 ROIs.

We then converted these 630-dimensional ROI data into average activations across the 7- and 17-dimensional Yeo et al. networks (Yeo et al., 2011). Each ROI was matched to the one network in the 7-network parcellation, and the one network in the 17-network parcellation, with which it had the highest overlap. We applied global signal regression to the 630 ROI data, averaged activity at each time point across all ROIs in each network, and finally *z*-scored activity in each network across the run. The resulting 7- and 17-dimensional data were then used for all subsequent analyses.

#### HCP.

Minimally preprocessed HCP data were downloaded from the March 2017 HCP 1200 Subjects Release. Data were then registered to the MNI152NLin6Asym space, run through a Gaussian smoothing kernel at 6-mm FWHM, and parcellated using the 512-region DiFuMo atlas (Dadi et al., 2020). Data were then detrended and low-pass filtered at 0.125 Hz, and several confounds—translational motion, rotational motion, framewise displacement, DVARS, white matter, and cerebrospinal fluid—were regressed out. For the 7- and 17-network data, as with the MyConnectome data, we first applied global signal regression, averaged activity at each time point across all ROIs in each of the 7 or 17 networks, and then *z*-scored the data. For the 512 ROI data, we followed this same procedure, skipping the step where we averaged across networks.

### Models

**Figure 11.**
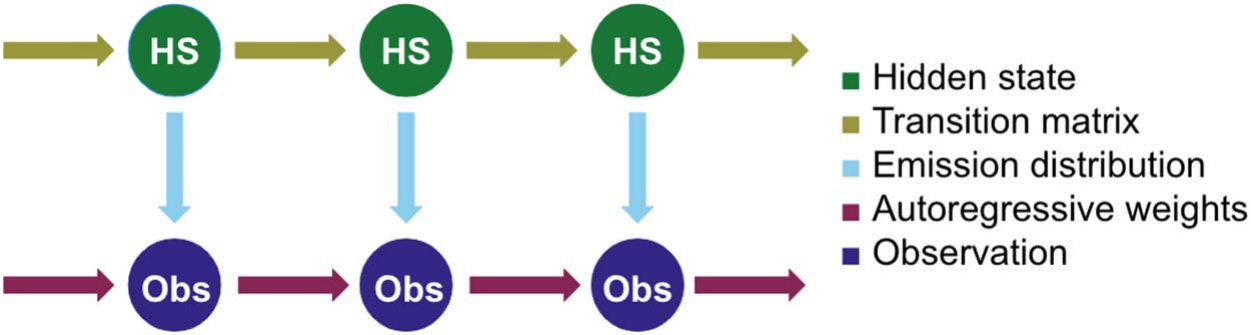
A schematic of the HMMs used in this paper. The Markov chain at the top of the image—the hidden states and the transition matrices—is common to all models, but the emission distributions vary based on the model type. The Gaussian models have no autoregressive weights.

All models were implemented using version 0.1.2 of the Dynamax library from Scott Linderman’s laboratory at Stanford University. The backbone of each model is a Markov chain with a prespecified number of hidden states (Figure 11). The transition probabilities among these *n* hidden states are stored in a transition matrix, which is learned based on the data. Each hidden state is associated with its own emission distribution, which helps to determine the observations. When these models are fit to data, the observations are set equal to the data.

To make this mathematically explicit, we will refer to the hidden state at time *t* as *z*_*t*_ and assume that the model has *K* hidden states *k*_1_, *k*_2_, …, *k*_*K*_. The observation at time *t* is an *N*-dimensional vector *y*_*t*_.

The probability that the hidden state at time *t* is *k*_*i*_, given that the hidden state at time *t* − 1 was *k*_*j*_, is simply

$$p\left(z_{t}=k_{i}|z_{t-1}=k_{j}\right)=T_{ji}$$ (1)

where *T* is the transition matrix. This equation holds true for all of the models used in this study.

Two different types of emission distributions are used in this study. For the Gaussian HMMs, the observation at time *t*, *y*_*t*_, follows the below probability distribution:

$$p\left(y_{t}|z_{t}=k_{i},\theta \right)=𝒩\left(y_{t}|\mu_{i},\Sigma_{i}\right)$$ (2)

where *μ*_*i*_ (the emission mean) is an *N*-dimensional vector, Σ_*i*_ (the emission covariance) is an *N* × *N*-dimensional diagonal matrix, and the emission distribution parameters *θ* = ${\left\{\mu_{k},\Sigma_{k}\right\}}_{k=1}^{K}$ (that is, a mean and covariance associated with each hidden state *k*).

For the ARHMM, observations follow the below emission distribution:

$$p\left(y_{t}|z_{t}=k_{i},y_{t-1},\theta \right)=𝒩\left(y_{t}|W_{i}y_{t-1}+b_{i},\Sigma_{i}\right)$$ (3)

where *W*_*i*_ is an *N* × *N* matrix of emission weights, *b*_*i*_ is an *N*-dimensional vector (the emission bias), Σ_*i*_ is an *N* × *N* matrix (the emission covariance), and the full set of parameters *θ* = ${\left\{W_{k},b_{k},\Sigma_{k}\right\}}_{k=1}^{K}$.

All models were initialized using the k-means algorithm: For a model with *K* hidden states, the data were assigned to *K* clusters, and the statistics of these clusters determined the initial values of some emission distribuion parameters. For Gaussian models, the means and covariances of the emission distributions were determined by the statistics of the clustered data. For autoregressive models, the biases of the linear function and the covariance were determined by the statistics of the clustered data, and the weights of the linear function were initialized to 0. Models were then trained using expectation maximization (EM).

### Training and Evaluation

#### MyConnectome.

**Figure 12.**
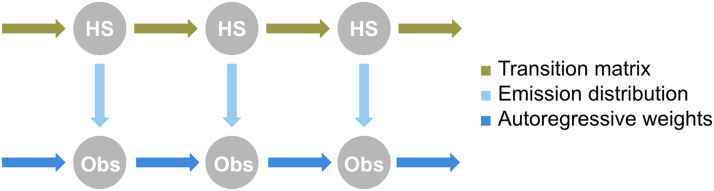
A schematic of the transition matrix-only training regime. Elements indicated in blue—the emission distribution and autoregressive weights—are trained on the full dataset, whereas the transition matrix, which is indicated in gold, is fine-tuned on the specific classes of training data. For the MyConnectome dataset, those classes are the caffeinated runs and the uncaffeinated runs. For the HCP dataset, each class is the set of runs from a single subject.

Models were trained according to two distinct regimes. In the “full” regime, all model parameters—initial hidden state probabilities, transition matrices, and emission distribution parameters like means, covariances, and autoregressive weights—were initialized and trained using the k-means and EM procedures. In the “transition-only” regime, however, an HMM of the desired type (i.e., autoregressive or Gaussian, with a particular number of hidden states) was first fit to the full dataset (except for the held-out data). Then, HMMs were partially retrained on specific subsets of the data (e.g., caffeinated vs. uncaffeinated, or runs from a specific individual): The emission distributions and initial probabilities were held constant, but the transition matrices were fine-tuned to individual subsets (again using EM) (Figure 12).

**Figure 13.**
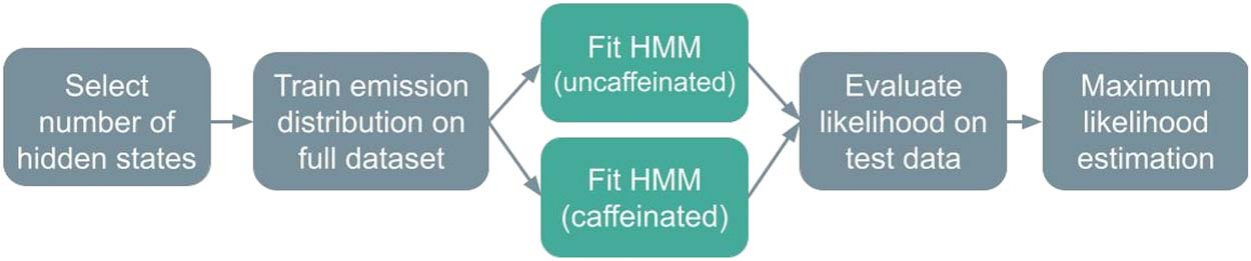
A schematic of the leave-one-run-out classification pipeline for MyConnectome data, in the transition matrix-only training regime.

Models were then evaluated for classification accuracy. At a high level, the classification scheme involved training a “menu” of models on specific subsets of the training data, then labeling each run from the test data according to its likelihood under each trained model. For the MyConnectome data, two different models were trained, one on a set of runs from “uncaffeinated” days and one on the same number of runs from “caffeinated” days. These models were then used to classify test runs. Each test run was classified as caffeinated or uncaffeinated by selecting the model under which those data had a higher log likelihood (Figure 13). Models were initially evaluated using leave-one-run-out cross-validation. During each iteration of cross-validation, a new “uncaffeinated” and a new “caffeinated” model were trained; these two models were always trained on the same number of runs, as a model trained on additional data would always have a likelihood advantage when evaluated on test data. Classification results from these models were then compared with a baseline linear SVM classifier trained on the functional connectivity matrices obtained from the same data, which was also evaluated using leave-one-out cross-validation. All classification accuracy results for the MyConnectome data were calculated as balanced accuracies. The full leave-one-out cross-validation procedure was run 100 times, as the model fitting is stochastic.

Finally, the classification approach was tested on seven uncaffeinated runs and five caffeinated runs that had been held out from the dataset throughout the process of implementing and testing the approach. Models were trained on the held-in data (26 caffeinated runs and 26 randomly selected uncaffeinated runs out of the 33 total uncaffeinated runs) before being evaluated on these held-out data. All analyses of held-out data were preregistered at https://osf.io/yv8f3.

The above analyses were run for all combinations of model emission distribution (Gaussian or autoregressive), training regime (full or transition only), and number of hidden states (2 to 12).

The time and computational resources required for model fitting and evaluation varied based on the specific model type (autoregressive or Gaussian, transition matrix-only or fully fit), the number of hidden states, and the dimensionality of the data (7- or 17-network). At the low end, one run of leave-one-out cross-validation for the transition matrix-only, Gaussian, two-hidden state model and the 7-network data took 31 s with 2 GB of memory. At the high end, one run of leave-one-out cross-validation for the fully fit, autoregressive, 12-hidden state model and the 17-network data took 14 min and 10 s with 2 GB of memory.

#### HCP.

**Figure 14.**
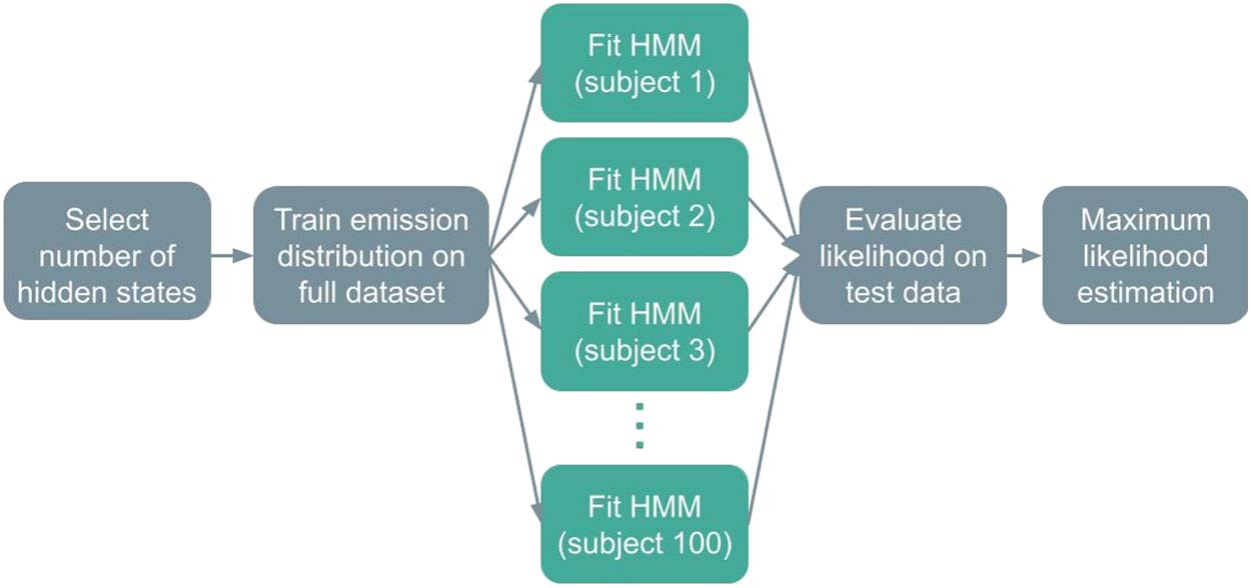
A schematic of the classification pipeline for HCP data, in the transition matrix-only training regime.

To evaluate the fingerprinting performance on the HCP data, a separate model was trained on three randomly selected runs of data from every subject, each of whom had four runs in total. Test data were classified as with the MyConnectome data—each run in the test dataset was labeled as the individual under whose model it had the highest log likelihood; but instead of comparing the test data’s likelihood under two different models, its likelihood was evaluated under 100 different models, one of which was trained on the other three runs from the same subject (Figure 14). In other words, to test the performance of this approach on a particular run, the other three runs from the same subject were used to train an HMM, and the log likelihood of the run under that HMM was compared with the log likelihood of that run under 99 other HMMs, each of which had been trained on a random subset of three runs from 1 of 99 other subjects. These 99 subjects were selected randomly from a pool of 149 possible subjects. Because the likelihood of the data was compared under 100 different models, the classification accuracy predicted by chance would be 1%. The random selection of 100 subjects and all subsequent fitting were repeated 100 times.

Classification results from these models were compared with a baseline “correlation of correlations” fingerprinting approach (Finn et al., 2015). The functional connectivity matrix for each run was calculated from the 7- or 17-network data. Three out of the four runs for each subject were randomly selected, and those functional connectivity matrices were averaged to create a set of 149 reference functional connectivity matrices. To label a given run of data from a particular subject, the three other functional connectivity matrices from that subject were averaged. Then, the Pearson correlation between the given run’s functional connectivity matrix and the averaged matrix was calculated, as well as its Pearson correlation with a set of 99 randomly selected reference connectivity matrices from other participants. The run was then labeled according to the matrix with which it had the highest correlation.

All fingerprinting approaches were evaluated on data from 47 subjects that had remained held out throughout the entire implementation and testing process. While model validation often does not involve any additional training, due to our classification scheme, we had to first train additional HMMs on each held-out subject before evaluating the performance of our method. However, our overall approach to HCP fingerprinting, and our implementation, was not modified after we began testing on the held-out data. When fingerprinting runs in the held-out data, HMMs or functional connectivity matrices calculated from that subject’s data were compared with HMMs/functional connectivity matrices calculated from 99 other randomly selected subjects, who may have been in the original or the held-out dataset. All analyses of held-out data were preregistered at https://osf.io/yv8f3.

The above analyses were run for all combinations of model emission distribution (Gaussian or autoregressive), training regime (full or transition matrix-only), and number of hidden states (2 to 12).

As with the MyConnectome task, the time and computational resources required for model fitting and evaluation varied based on the specific model type (autoregressive or Gaussian, transition matrix-only or fully fit), the number of hidden states, and the dimensionality of the data (7- or 17-network). At the low end, one run of fingerprinting (which involves predicting subject identity for 400 different rsfMRI runs) for the transition matrix-only, Gaussian, two-hidden state model, and the 7-network data took 1 min and 33 s with 2 GB of memory. At the high end, one run of fingerprinting for the fully fit, autoregressive, 12-hidden state model, and the 17-network data took 38 min and 12 s with 4 GB of memory.

### Model Features

After the models were fit to the MyConnectome data and their classification performance was evaluated, the dynamical features of the models were investigated in more detail. First, the transition matrices of ARHMMs fit to uncaffeinated and caffeinated runs were examined. These transition matrices were generated using the transition matrix-only fitting procedure, in which the emission distributions (including autoregressive weights) were first fit to the entire dataset of both caffeinated and uncaffeinated runs, and then the transition matrices were separately fine-tuned on either all of the uncaffeinated runs or all of the caffeinated runs. The caffeinated and uncaffeinated transition matrices were then compared.

Next, occupancy times for the hidden states were examined. Given a model and a run of data (or in terms of the model, a sequence of observations), a hidden state associated with each of those observations can be inferred by calculating the maximum a posteriori estimate of the sequence of hidden states, given the data. In this case, the model used was an ARHMM that had been fit to the entire dataset, and the data were all of the caffeinated and uncaffeinated runs (except those that had been held out) from the MyConnectome dataset. The sequence of hidden states associated with each run was calculated using the Viterbi algorithm (Forney, 1973). For each run and each hidden state, occupancy was calculated as the fraction of the total run spent in that hidden state. Average network activity in each hidden state was calculated by averaging the activity across all of the time points from all runs that were labeled with that hidden state by the Viterbi algorithm.

### Evaluating the Impact of Motion and Family Structure

To evaluate whether head motion may have driven classification performance on the MyConnectome data, we directly compared the performance of models that were trained on data where high-motion volumes had been censored and interpolated over with models trained on data without interpolation. First, we generated a motion-free dataset by collecting all continuous sequences of motion-free volumes over 10 volumes in length in the MyConnectome dataset. Because HMMs only use the hidden state at time *t* − 1 to predict the hidden state at time *t*, it was important to consider each of these continuous sequences to be its own separate run. We then generated another dataset that had exactly this same structure (i.e., same number of “runs,” with the same run lengths) from the interpolated MyConnectome data. This resulted in two datasets—one with only low-motion volumes and one with both low-motion and interpolated volumes.

We then tested the classification performance on both of these datasets. Because this approach resulted in time series of different lengths, and Dynamax is not able to fit models to such data, we performed this analysis using the Dynamax’s precursor library, ssm. ssm does not natively allow for transition matrix-only training, so we only tested the fully trained classification approaches using this technique. This analysis was performed using the 7-network data.

We also examined whether the family structure present in the HCP dataset affected the HMM-based fingerprinting performance. To conduct this analysis, we randomly selected 100 of the 149 individuals in the primary HCP sample and tested our approach for classifying each of the four runs from those individuals as described above. Whenever our approach incorrectly classified a run (that is, the run had the highest likelihood under the model trained on a different subject’s data), we recorded that instance in our confusion matrix, which was keyed both to the subject from whom the run who had originated and the subject under whose HMM that run had the highest log likelihood. Then, we counted up the number of confusions that occurred between family numbers and divided it by the total number of confusions to obtain an “error fraction.” To generate a null distribution for our error fraction, we repeated this analysis with family membership labels randomly permuted. For each number of hidden states and model type, we repeated the unpermuted analysis five times and the permuted analysis 20 times. This analysis was performed using the 7-network data.

## ACKNOWLEDGMENTS

Data were provided in part by the Human Connectome Project, WU-Minn Consortium (Principal Investigators: David Van Essen and Kamil Ugurbil; 1U54MH091657) funded by the 16 NIH Institutes and Centers that support the NIH Blueprint for Neuroscience Research and by the McDonnell Center for Systems Neuroscience at Washington University.

## SUPPORTING INFORMATION

Supporting information for this article is available at https://doi.org/10.1162/netn_a_00412.

## AUTHOR CONTRIBUTIONS

Grace Huckins: Conceptualization; Formal analysis; Funding acquisition; Investigation; Methodology; Software; Validation; Visualization; Writing – original draft; Writing – review & editing. Russell Poldrack: Conceptualization; Data curation; Methodology; Project administration; Resources; Supervision; Writing – review & editing.

## DATA AND CODE AVAILABILITY STATEMENT

All code and network-averaged data are available at https://github.com/ghuckins/HMMMyConnectome.

## Supplementary Material



## TECHNICAL TERMS

Hidden Markov model: A class of dynamical model where, at time *t*, the model is always in one of *n* hidden states, and the hidden state at time *t* + 1 only depends on the hidden state at time *t*. Each hidden state is associated with an emission distribution.
Emission distribution: For each hidden state in a hidden Markov model, the emission distribution specifies the distribution from which observations are drawn while the model is in that hidden state.
Fingerprinting: Identifying an individual solely on the basis of their fMRI data.
Transition matrix: An *n* × *n* matrix that specifies the probability of transitioning from any of the *n* hidden states to any of the *n* hidden states when the hidden Markov model progresses forward one timestep.
Support vector machine: A simple classification approach in machine learning that works by finding the optimal hyperplane separating two datasets.
Cross-validation: An approach to evaluating the performance of a supervised model, where the dataset is divided into *n* folds and the model is iteratively trained on each subset of *n* − 1 folds before being tested on the remaining fold.
Generative classifier: A classifier that labels data by evaluating the likelihood of those data under a set of different models and identifying the model under which the data’s likelihood is maximized.
Autoregressive model: A dynamical model where the value of the variable at time *t* is a linear combination of its values at previous times, plus noise.
Expectation maximization: An iterative algorithm used to estimate optimal parameter values in order to fit some statistical models to data.
