# Supplementary material for: Generative dynamical models for classification of rsfMRI data
Source: Netw Neurosci. 2024 Dec 10;8(4):1613–33. doi: 10.1162/netn_a_00412 (PMC11675094; doi:10.1162/netn_a_00412)
Supplement: Supplementary file 1 [file netn-8-4-1613-s001.pdf]

Supplementary Information

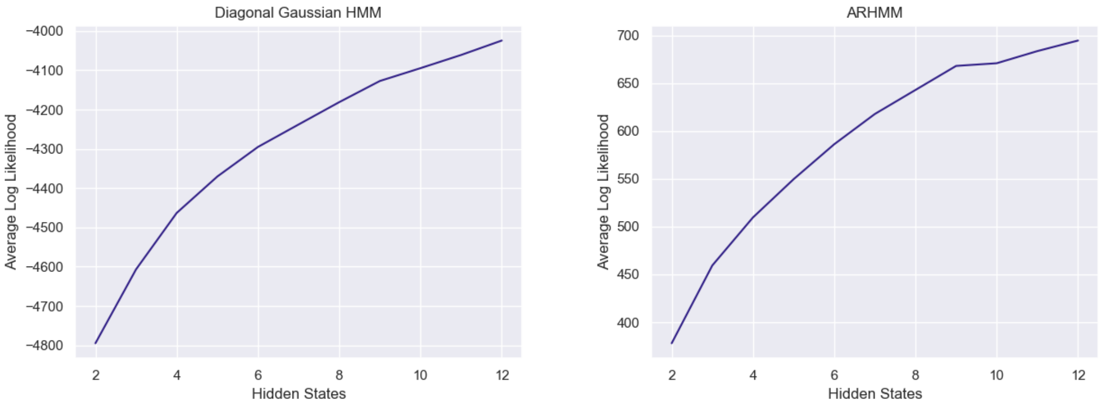

Figure 1: The cross-validated log likelihood of the MyConnectome data under both a diagonal Gaussian HMM (left) and ARHMM (right). Log likelihood was calculated using 5-fold cross-validation.

	**Coefficient**	**Standard Error**	t	p
Intercept	0.759	0.003	285	< 0.001
Model: Autoregressive, Full	0.0096	0.002	4.02	< 0.001
Model: Gaussian, Full	-0.183	0.002	-76.5	< 0.001
Model: Gaussian, Trans Only	-0.234	0.002	-98.1	< 0.001
Networks	-0.0031	0.002	-1.84	0.066
Hidden States	0.0060	0.000	22.4	< 0.001

Table 1: Linear regression on the MyConnectome cross-validation results, with classification accuracy as the dependent variable. Each model is being compared against the "Autoregressive, Trans Only" model. When considered together, all "Model" variables also have a p value < 0.001. This last p value was calculated by comparing the likelihood ratio of the regression model fit above with the likelihood ratio of a regression model that did not include "Model" as a predictor. Linear regression was performed using the statsmodels package.

	**Coefficient**	**Standard Error**	t	p
Intercept	0.157	0.004	40.5	< 0.001
Model: Autoregressive, Full	0.409	0.004	114	< 0.001
Model: Gaussian, Full	0.269	0.004	75.4	< 0.001
Model: Gaussian, Trans Only	-0.0265	0.004	-7.24	< 0.001
Networks	-0.150	0.003	-59.7	< 0.001
Hidden States	0.0007	0.000	1.78	0.075

Table 2: Linear regression on the Human Connectome Project cross-validation results, with classification accuracy as the dependent variable. Each model is being compared against the "Autoregressive, Trans Only" model, When considered together, all "Model" variables also have a p value < 0.001. This last p value was calculated by comparing the likelihood ratio of the regression model fit above with the likelihood ratio of a regression model that did not include "Model" as a predictor. Linear regression was performed using the statsmodels package.
